# Evaluating Deep Q-Learning Algorithms for Controlling Blood Glucose in In Silico Type 1 Diabetes

**DOI:** 10.3390/diagnostics13193150

**Published:** 2023-10-07

**Authors:** Miguel Tejedor, Sigurd Nordtveit Hjerde, Jonas Nordhaug Myhre, Fred Godtliebsen

**Affiliations:** 1 Norwegian Centre for E-Health Research, P.O. Box 35, N-9038 Tromsø, Norway; miguel.tejedor@ehealthresearch.no; 2 Faculty of Science and Technology, Norwegian University of Life Sciences, Postboks 5003 NMBU, 1432 Ås, Norway; sigurdhjerde@protonmail.com; 3 NORCE Norwegian Research Centre, Postboks 22, Nygårdstangen, 5838 Bergen, Norway; jonm@norceresearch.no; 4 Department of Mathematics and Statistics, UiT—The Arctic University of Norway, P.O. Box 6050 Langnes, N-9037 Tromsø, Norway

**Keywords:** reinforcement learning, type 1 diabetes, Q-learning, deep learning, artificial pancreas

## Abstract

Patients with type 1 diabetes must continually decide how much insulin to inject before each meal to maintain blood glucose levels within a healthy range. Recent research has worked on a solution for this burden, showing the potential of reinforcement learning as an emerging approach for the task of controlling blood glucose levels. In this paper, we test and evaluate several deep Q-learning algorithms for automated and personalized blood glucose regulation in an in silico type 1 diabetes patient with the goal of estimating and delivering proper insulin doses. The proposed algorithms are model-free approaches with no prior information about the patient. We used the Hovorka model with meal variation and carbohydrate counting errors to simulate the patient included in this work. Our experiments compare different deep Q-learning extensions showing promising results controlling blood glucose levels, with some of the proposed algorithms outperforming standard baseline treatment.

## 1. Introduction

**Figure 1:**
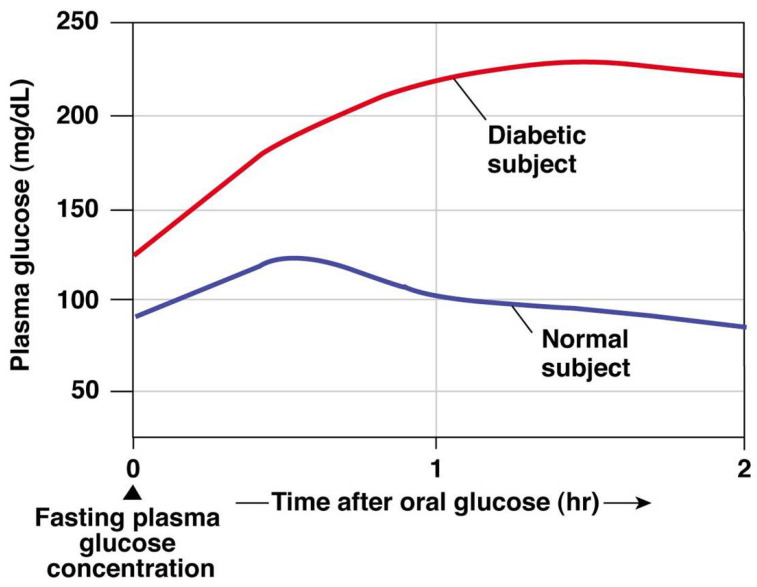
Glucose tolerance test for diagnosis of diabetes: healthy and diabetic subjects [6].

Diagnoses of blood sugar conditions are determined by the insulin secretion from the pancreas [1]. In this regard, type 1 diabetes (T1D) is a chronic disease that occurs when the pancreas is no longer able to produce enough insulin because of the autoimmune destruction of insulin-producing beta cells in the pancreas [2]. This metabolic disorder leads to high blood glucose (BG) levels (hyperglycemia), causing damage, dysfunction, and failure of various organs in the long term [1]. T1D treatment consists of regulating BG levels using external insulin doses, whereas administering more insulin than needed might cause dangerous low BG levels (hypoglycemia) [3]. The fear of hypoglycemia is a major concern for most T1D patients, since it can be fatal if unnoticed [4]. The goal of the treatment is to maintain BG levels in a healthy target range between 70 and 180 mg/dL, referred as normoglycemia [5]. Figure 1 shows the results from a glucose tolerance test where the BG values from a healthy subject and a diabetic subject are compared. In this test, oral glucose is given to the subjects and blood samples are taken afterward to determine BG clearance. This test is usually used in diabetes diagnosis, since diabetic BG rises to hyperglycemic values due to the lack of insulin.

Insulin is subcutaneously administered by the patients either through multiple daily injections or an insulin pump providing a continuous infusion [7]. Patients under multiple daily injections treatment follow a basal–bolus insulin regimen, taking a basal long-acting insulin dose approximately once a day to regulate fasting BG levels, and short-acting insulin boluses at mealtimes to reduce the effect of carbohydrate intake. Alternatively, the insulin pump continuously delivers short-acting insulin as a basal rate, whereas boluses are manually activated by the patient to deal with high BG levels associated with meal intakes. In addition, BG levels have to be monitored by the patients either several times per day using manual finger-prick measurements, or using a continuous glucose monitor (CGM) embedded in the subcutaneous tissue [8].

The artificial pancreas (AP) is the combination of an insulin pump, a CGM, and a control algorithm to automatically regulate BG concentrations [9,10]. The control algorithm translates BG levels measured by the CGM into the insulin amount to be delivered by the pump. The subcutaneous administration of insulin causes a delay in the insulin action, whereas subcutaneous BG measurements from the CGM are also delayed. Apart from the insulin action and CGM delays, the dynamic factors causing variation in the patient-specific parameters, the non-stationary daily disturbances, and the noisy data from the sensors provide a challenging control problem complicating the achievement of healthy BG levels [11,12]. The commercial available AP systems [13,14,15,16], do-it-yourself systems [17], and academic systems [18] are all hybrid closed-loop systems. A hybrid system fully automates the basal insulin deliveries, whereas the patient has to provide information about carbohydrate ingestion to calculate boluses during meals.

The requirement for an adaptive algorithm that personalizes the system for each patient is one of the major limitations of the AP [11]. The state-of-the-art in AP controller algorithms consists mainly of either proportional–integral–derivative control [15,19], or model predictive control [20,21]. Traditional reactive controllers based on momentary BG changes cannot thus keep up with the delays inherent to the AP systems to avoid hyperglycemic events after meals. In addition, the variability in BG concentration due to meal intake, exercise, sleep, and stress are not yet modeled efficiently [10], hindering the development of an adaptive AP. The control algorithm should be able to learn rich enough models that adapt to the system as a whole [11], encouraging the use of model-free approaches. At this stage, reinforcement learning (RL) has emerged as a promising alternative to traditional paradigms for controlling insulin infusion in the AP [22].

RL algorithms have been used before to regulate the BG levels in in silico T1D patients, showing that RL algorithms can improve BG control. Concretely, in Sun et al. [23], RL is used to learn the insulin-to-carb ratio parameter of the insulin pump, but not the insulin action itself. In Fox and Wiens [24], the performance of some RL methods is compared to a proportional–integral–derivative algorithm. Lee et al. [25] used a proximal policy optimization method for automated insulin infusion with a reward function that mimics the natural behavior of the missing beta-cells. Zhu et al. [26] propose a Q-learning approach where the basal rate is modified by a discrete number of actions. They also operate in a dual-hormone approach with glucagon infusion as one of the actions. In Yamagata et al. [27], model-based RL combining echo state networks and a model predictive controller is proposed for blood glucose control. Emerson et al. [28] evaluate the use of offline RL methods to control blood glucose levels and avoid potentially dangerous patient interaction during the training process. Viroonluecha et al. [29] propose deep RL approaches for blood glucose control in a closed-loop system with a reduced frequency of observations and rewards. For an extensive review of the role of RL and its applications in healthcare, refer to [30,31]. Machine learning techniques have been also used in diabetes diagnosis and screening [32]. Khaleel et al. [33] propose a machine learning model to predict whether a patient has diabetes or not.

In this work, we implement and evaluate several state-of-the-art improvements to the deep Q-learning (DQL) algorithms in the hybrid closed-loop AP to automatically regulate the BG levels in a T1D patient. We perform in silico experiments using the Hovorka model [20], demonstrating that RL can adapt to carbohydrate counting errors and in some cases outperform traditional basal–bolus treatment. We compare the performance of the different DQL extensions in terms of time-in-range (time spent on healthy BG levels), time in hypo-/hyperglycemia, and BG level plots for visual inspection. This work is based on the Master’s thesis of Sigurd Nordtveit Hjerde [34].

### Structure of Paper

We begin introducing RL and the T1D simulation environment in Section 2. In Section 3 we present the results. In Section 4 we discuss the results of this work. Section 5 provides concluding remarks and directions from possible future work.

## 2. Methods

In this section, we introduce the RL framework, the deep Q-learning algorithm and its extensions, and the T1D simulator used in this work.

### 2.1. Reinforcement Learning

**Figure 2:**
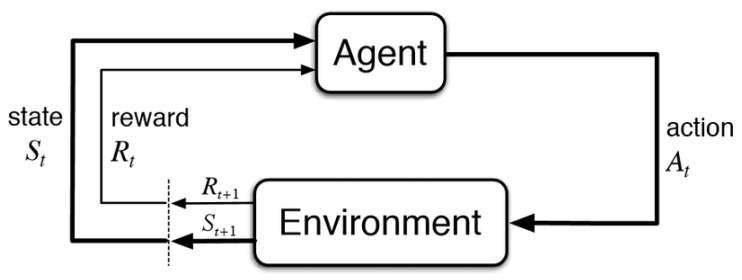
The reinforcement learning framework [35].

RL is characterized by the interactions between a decision-making *agent* and its unknown *environment*. This framework is shown in Figure 2, where at each time step the agent perceives the current *state* of the environment and takes an *action* based on that state. As a consequence of this action, the environment moves to a new state and generates a positive or negative *reward* for the agent. The goal of the agent is to maximize the reward in the long run by taking actions that result in preferable states [35].

A RL problem can be formulated as a Markov decision process represented by the tuple $\left(S,A,P,R,\gamma \right)$, where S and A are the state and the action spaces, respectively. P are the state transition probabilities $p(s^{\prime }|s,a)$, representing the transition from state *s* to $s^{\prime }$ when the agent takes action *a*. R represents the numerical rewards from reward function $r(s,a,s^{\prime })$, which defines the goal of the problem, and γ∈(0,1) is the discount factor. The *policy* represents the mapping from state to action and the goal of the agent is to learn an optimal policy $\pi^{*}$ that maximizes the accumulated reward over time represented by the expected return $G_{t}=\sum_{k=0}^{\infty }\gamma^{k}R_{t+k+1}$, where $R_{t}=r\left(s_{t},a_{t},s_{t+1}\right)$. The total amount of reward expected by the agent starting from the state *s* and thereafter following the policy π is called the value function $V_{\pi }\left(s\right)$, which represents the long-term desirability of states:

$$V_{\pi }\left(s\right)=E_{\pi }\left[G_{t}|S_{t}=s\right]=E_{\pi }\left[\sum_{k=0}^{\infty }\gamma^{k}R_{t+k+1}|S_{t}=s\right].$$

Similarly, the total amount of reward expected by the agent starting from the state *s*, taking action *a*, and thereafter following the policy π is called the action–value function $Q_{\pi }\left(s,a\right)$:

(1) $$\begin{array}{cc} Q_{\pi }\left(s,a\right) & =E_{\pi }\left[G_{t}|S_{t}=s,A_{t}=a\right] \end{array}$$

(2) $$\begin{array}{cc} \; & =E_{\pi }\left[\sum_{k=0}^{\infty }\gamma^{k}R_{t+k+1}|S_{t}=s,A_{t}=a\right]. \end{array}$$

### 2.2. Q-Learning

The goal of RL is to find an optimal policy that is better than or equal to all other policies based on the values of the states. Actions are then taken such that the agent spends as much time as possible in valuable states. The policy is often simply a greedy search over each action in the given state, where the action that gives the highest value is chosen [36]. In the case of an agent controlling insulin infusion in T1D, safe blood glucose levels would represent states with high values, whereas high and low blood glucose levels would be represented by states with lower values.

One of the most popular methods to estimate the Q-values is the Q-learning algorithm [37], where the action–value function Q(s,a) is learned through temporal-difference updates [35]. Assuming finite state and action spaces, the Q-learning algorithm is given by

(3) $$Q_{i+1}\left(s_{t},a_{t}\right)=Q_{i}\left(s_{t},a_{t}\right)+\alpha \left[r_{t+1}+\gamma \max_{a}Q_{i}\left(s_{t+1},a\right)-Q_{i}\left(s_{t},a_{t}\right)\right],$$

where α∈(0,1] is the learning rate. The Q-values will converge to an optimal action–value function $Q^{*}$, where the optimal policy $\pi^{*}\left(s_{t}\right)={arg max}_{a\in A}Q\left(s_{t},a\right)$ can be extracted [37].

#### 2.2.1. Q-Learning Extensions

In this section, we briefly introduce the Q-learning extensions used in this work. References to the original papers are included for a full description of the algorithms.

- Deep Q-Learning (DQN): In DQN, the Q-value function is approximated using a neural network (NN). The input of the NN is the current state of the environment, whereas the output is the Q-value of all the possible actions the agent can take [38].
- Double Q-Learning (DDQN): This approach proposes two Q-value approximators represented by two NNs, one to estimate the target Q-values and the other one to estimate the predicted Q-values. DDQN reduces the overestimation of the Q-values, usually leading to a better performance compared to DQN [39].
- Dueling DQN and DDQN: This extension changes the standard DQN and DDQN architectures presenting two separate estimators, one for the state-value function and one for the advantage function. The advantage function is defined as the difference between the Q-value function and the state-value function, A(s,a)=Q(s,a)−V(s), and indicates the amount of reward that could have been obtained by the agent by taking the action *a* over any other action. This method increases the stability of the optimization [40].
- Prioritized Experience Replay (PR): DQN training is not efficient since transitions are randomly sampled to train the network parameters. PR gives priority measures to the transitions, sampling important transitions more frequently [41].
- Noisy DQN: Standard DQN uses a random policy resulting in an inefficient exploration, whereas noisy DQN adds parametric noise to the weights and biases for exploration [42].
- Categorical DQN: This algorithm learns a distribution of the Q-values instead of an estimation of the Q-value function, leading to a more stable and faster learning and usually outperforming standard DQN [43].
- Rainbow DQN: This algorithm integrates all the previous extensions introduced in this section, improving data efficiency and overall performance [44].

### 2.3. In Silico Simulation

There exist three main physiological models in in silico T1D research: the Bergman minimal model [45], the Hovorka model [20], and the UVA/Padova model [46,47]. The Bergman minimal model includes only two equations describing the internal insulin and glucose dynamics, with no delays associated with the subcutaneous insulin infusion and glucose measurements. Despite its simplicity, the minimal model glucose kinetics is still widely used in diagnosis as a clinical tool to calculate insulin sensitivity index [48]. The Hovorka and the UVA/Padova models both account for these significant delays. The Hovorka model consists of five compartments describing the insulin action and glucose kinetics dynamics [49]: three internal compartments describing insulin action, glucose kinetics, and glucose absorption from the gastrointestinal tract, and two external compartments describing interstitial glucose kinetics and subcutaneous insulin absorption. We use the Hovorka model in this work, which includes the virtual patient used in our experiments.

#### 2.3.1. Experiment Setup

Three experiments were included in this work. The first experiment compares all the DQN algorithms introduced in Section 2.2.1, while still using the same hyperparameters, training duration, and batch size. The second experiment includes the same algorithms from the first experiment, but uses a larger action space to explore how more actions affect the performance of the RL agents. Lastly, the third experiment was organized to test how well a trained agent would perform when skipping meal boluses at random.

State-of-the-art AP designs utilize commercially available insulin pumps and CGMs, operating in the subcutaneous tissue and introducing serious delays into the control task [50]. The hybrid closed-loop AP proposed in this work utilizes subcutaneous devices with short-acting insulin, which starts to work after 30–60 min and peaks after around 2–4 h. This implies that the actions from an agent would not be immediately be reflected by the CGM measurements and the state of the environment would not be well represented by only including BG data [51]. In this work, we have included insulin information as part of the state representation and considered 30 min time intervals as the time between each updated state from the environment to alleviate the effect of the delays in the learning process. Therefore, the insulin basal rate is kept constant during these 30 min and the environment has enough time to significantly change between each time step.

The states $s_{t}\in S$ consist of the previous 30 min of BG data, as well as the 4 last insulin actions (last 2 h) at a time resolution of 1-min: $s_{t}=\left[G_{t},I_{t}\right]$, where $G_{t}=\left[g_{t-29},g_{t-28},\cdots ,g_{t}\right]$ and $I_{t}=\left[i_{t-3},i_{t-2},i_{t-1},i_{t}\right]$, with $g_{t}\in R_{0:500}$, $i_{t}\in R_{+}$ and $t\in N_{0:72}$. Here, $g_{t}$ [mg/dL] are the BG measurements, $i_{t}$ [mU/min] are the insulin basal rates, and *t* is the time index, where one time step is 30 min. The time step limit is at 72 because we simulate the patient using episodes of 1.5 day =36 h =2160 min, and divided by the 30 min step, we obtain 2160/30=72. This is for taking into account the whole night before the next day.

**Figure 3:**
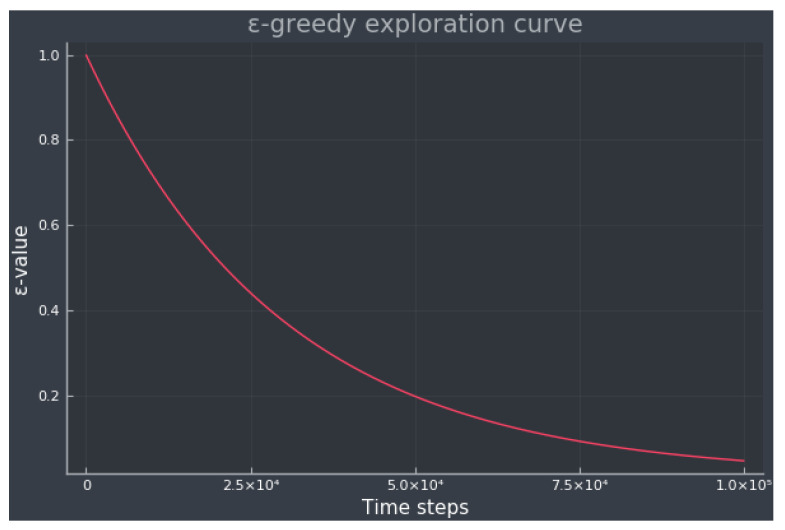
The ε-greedy exploration curve for all experiments. The ε-value shows the percentage of exploration at the current time step.

The agent performs an action $a_{t}\in A$ every *t* steps, i.e., every 30 min. We define $a_{t}$ as real positive numbers $R_{+}$ in a discrete action space. In this work, we used two different action spaces: $A_{1}=\left\{b_{0},b^{*},3b^{*}\right\}$ and $A_{2}=\left\{b_{0},b^{*}/2,b^{*},2b^{*},3b^{*}\right\}$, where $b_{0}$ is 0 insulin (stop the insulin pump) and $b^{*}$ [mU/min] is the optimal basal rate, which is set to 6.43 mU/min. The optimal basal rate is calculated as the minimum amount of insulin required to manage normal daily BG fluctuations and keep the BG level at target value during the steady state for this particular patient. Note that both action spaces have the same minimum and maximum actions, but the $A_{2}$ has a higher resolution including two more actions in between. Decaying ε-greedy exploration was used during training for all experiments, according to equation:

(4) $$\varepsilon \left(t\right)=\varepsilon_{F}+\left(\varepsilon_{0}-\varepsilon_{F}\right)e^{-t/\eta },$$

where the initial value was set to $\varepsilon_{0}=1.0$, the final value $\varepsilon_{F}=0.01$, and the decay $\eta =3\times 10^{4}$. The exploration curve can be seen in Figure 3, and is approximately equivalent to 50% exploration during training.

We obtain a new state $s_{t}$ every 30 min, and when the agent performs an action $a_{t}$ we receive the next state $s_{t+1}$ and a reward $r_{t}\in R\subset R$. The reward function *R* is defined as a Gaussian function:

(5) $$R\left(g_{t}\right)=e^{-\frac{1}{2}{\left(g_{t}-b_{r}\right)}^{2}/900},$$

where $g_{t}$ is the BG level and $b_{r}$ [mg/dL] is the BG reference, which is set to 108 mg/dL. In addition to this, the simulator checks if the BG levels are within valid bounds, i.e., $\left[g_{\ell },g_{h}\right]$ [mg/dL], where $g_{\ell }=70$ mg/dL is the lower bound and $g_{h}=180$ mg/dL is the higher bound. If $g_{t}\in \left[g_{\ell },g_{h}\right]$, then $r_{t}>0$. Otherwise, the agent receives a reward of $r_{t}=-1000$, which can be interpreted as a punishment.

To measure the performance of our simulations, we use the time-in-range (TIR), which is the percentage of time the patient spends with its BG levels within the target range, defined as the healthy BG range between 70 and 180 mg/dL [52]. This performance measurement can also be perceived as the number of hours per day spent within the desired target range. As an example, 12 h per day spent within the target range correspond to 50% TIR. Now, consider andincrease from 50% TIR to 55% TIR. This 5% increase translates to one more hour per day spent within the target range, which is a significant increase considering the small change in the TIR. We also define the metrics time-above-range (TAR) and time-below-range (TBR) as the percentage of time the patient spends with its BG levels above and below the TIR, respectively. Finally, we included the mean BG per episode, μ, and the standard deviation of the BG per episode, σ.

An individual who weighs 70 kg was used during the experiments. For each training episode, the virtual patient was given meals from a random meal generator. Fixed seed was used to ensure each agent trained on the same dataset.

The meal schedule was defined as follows: 4 meals per day with a set schedule lasting up to 1 min. Uniform noise, v∼U(−20,20), was added to each base meal to simulate meal variation, as well as ±30 min to each meal time. The daily meal schedule is then:

- Breakfast: (40 + $v_{1}$) [g] of CHO at 8:00 ± 30 min.
- Lunch: (80 + $v_{2}$) [g] of CHO at 12:00 ± 30 min.
- Dinner: (60 + $v_{3}$) [g] of CHO at 18:00 ± 30 min.
- Supper: (30 + $v_{4}$) [g] of CHO at 22:00 ± 30 min.

Here, $v_{1}$, $v_{2}$, $v_{3}$, and $v_{4}$ are the four noise variables, one for each meal. The base meals (40 g, 80 g, 60 g, and 40 g) of carbohydrates are taken from El Fathi et al.’s work [5]. Each meal consists of an actual CHO intake and an estimated CHO intake. The estimated intake is used for meal bolus calculation and is included in both training and testing to simulate ±30% carbohydrate counting error of the actual intake. To test the agents, we use a fixed set of 100 episodes with 100 daily meal scenarios, sampled from the meal generator with a different seed than the training meals.

Four different NN architectures were used in our experiments. The architectures assigned to each of the algorithms are described below:

- DQN and DDQN: A 4 layer fully connected network with 64 hidden units each. ReLU nonlinearity was used across all layers. The output layer has a linear output.
- Dueling DQN, dueling DDQN, PR DQN, and noisy DQN: A fully connected network consisting of two blocks, each individual block, is similar to the DQN network. Each output layer in the two blocks has linear outputs, representing the advantage and value streams. For the noisy DQN algorithm, we simply added noise to the linear layers and reset the noise parameters after every training batch.
- Categorical DQN: A 4 layer fully connected network with 64 hidden units each. The two first layers are without noise and the following are with noise. ReLU nonlinearity was used across all layers. The output layer has a linear output.
- Rainbow DQN: A fully connected network consisting of two blocks, each with a dense layer of 3 fully connected noisy layers. The input layer consists of an additional linear layer before splitting into the two streams. The amount of hidden units is 64, and ReLU nonlinearity was used across all layers. Each output layer is linear and represents the advantage and value streams as in the dueling DQN case.

Algorithms and NN implementations were conducted in Python 3.8.1 using PyTorch 1.4 [53]. The full code is available at repository https://github.com/sigurdhjerde/Masters-Thesis. The in silico simulator was wrapped in the OpenAI Gym framework for simplified testing [54], and its implementation can be viewed at repository https://github.com/sigurdhjerde/gym/tree/master_student_branch. The mean squared error training loss of the TD errors was optimized using Adam, with a learning rate of $10^{-3}$. Our NN weights and biases were initialized using PyTorch default settings.

## 3. Results

The main goal of the following experiments is to compare different DQN extensions for the BG simulations, in which the diabetic patient should maximize the TIR while minimizing the TAR and TBR. Different action space sizes will also be compared, as this might affect the performance of the algorithms. The baseline refers to the patient using a fixed basal rate with the optimal value $b^{*}=6.43$ mU/min. This baseline will serve as a guideline when comparing trained RL agents for different DQN extensions. Note that the baseline performance is already quite high and not realistic for our in silico patient, but the results are still very valuable since we can still monitor the performance of the different RL algorithms.

### 3.1. Experiment 1—Comparing Algorithms

In this experiment, we compare all the DQN algorithms introduced in Section 2.2.1. The main goal here is to see which algorithm achieves the best TIR score calculated from the mean BG per minute over 100 episodes. We used the state space and the action space with three actions as described in Section 2.3.1. The models were trained for $10^{5}$ time steps, with a batch size of 128, an experience replay buffer size of $10^{5}$, and a discount factor of γ=0.99. The results are summarized in Table 1.

These results show that it is possible to control BG levels using RL in the proposed experimental setup. Analyzing and comparing the presented DQN extensions, we found different levels of performance. The DQN algorithm performs very similarly to the baseline in terms of the TIR, TAR, and TBR. The standard deviation of the BG per episode σ (35.21) is slightly higher for DQN because the agent is not able to properly compensate for larger meals, scoring the worst out of all the tested algorithms. We see that in general, the σ values do not vary much. This can be perceived as that for each episode, the variation in BG does not differ that much from algorithm to algorithm.

The DDQN algorithm presents a low TAR (1.90%) because the agent tends to choose higher basal rate actions. As a consequence of the higher basal rates, the TBR increases (5.28%) compared to baseline (0.0%), whereas the TIR and the mean BG per episode decrease, presenting the worst results in terms of the TIR (92.82%) and the closest μ = 111.67 mg/dL to the BG target value.

Regarding dueling DQN, we observe a TIR estimate approximately 2% lower than the baseline TIR, whereas the TAR has also decreased (0.45%). The TBR estimate is higher, scoring the highest TBR with a percentage of 6.25%. Similar to the DDQN algorithm, the low performance achieved by dueling DQN is the result of a set of actions with a high basal rate.

The dueling DDQN algorithm shows better performance than the baseline, presenting a higher TIR (96.71%) and lower TAR (3.29%) while keeping the 0.0% TBR. The overall BG is higher per episode, as we can see from the μ estimate (126.92 mg/dL), scoring the highest value among the different tested algorithms. This algorithm proves to be competitive when controlling BG levels in a simulated T1D patient.

The PR DQN agent presents the highest TAR (5.65%), scoring the worst among the different algorithms. In addition, the TIR for this agent is 94.35%, not improving the results obtained from the baseline (95.41%).

The noisy DQN algorithm obtains the best results in terms of the TIR (97.04%) and BG standard deviation per episode σ= 31.74 mg/dL. This agent also presents a low TAR (2.96%) and a good mean BG per episode μ = 116.10 mg/dL, considering that TBR is kept to 0.0%. Noisy DQN outperforms the baseline with lower episodic BG levels and no hypoglycemic events, emerging as our best solution for controlling BG concentrations.

Similar to DQN, the categorical DQN extension follows a strategy close to the baseline, obtaining results very similar to the baseline and DQN methods. This agent presents a slightly lower TIR and higher TAR, mean, and standard deviation BG than the baseline.

Rainbow DQN drops BG levels due to higher basal rates, leading to the lowest episodic BG with mean BG per episode μ = 100.66 mg/dL, TAR = 0.0%, and the second worst TBR = 5.56%. The TIR = 94.35% is lower than the baseline (95.41%), while still able to avoid hypoglycemic events.

**Figure 4:**
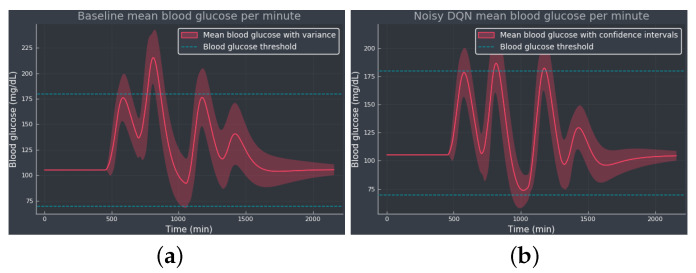
The mean BG per minute with confidence bands (shaded red area), representing the standard deviation, simulated for 100 episodes: (**a**) The baseline using only the optimal basal rate $b^{*}=6.43$ mU/min as the selected action. (**b**) The noisy DQN agent. The blue dotted lines indicate the normoglycemic range fixed at 70–180 mg/dL.

Figure 4 shows the mean BG values obtained from baseline and noisy DQN agent, considered the most competitive RL approach from the results obtained in Table 1. Comparing both methods, we see how the noisy DQN agent outperforms the baseline, increasing the TIR by reducing the hyperglycemic events while avoiding hypoglycemia. Note how the agent regulates the tail of the curve to the optimal BG value, showing that the agent was able to learn the optimal basal rate of our simulated patient. The RL algorithm achieves an overall reduction in the mean BG values and the standard deviation.

### 3.2. Experiment 2—Expanded Action Space

The goal of this experiment is to see what influence an increase in the action space has on the agent. By increasing the action space, one would assume that learning the right action at a certain state could be more difficult. If learned right, more actions could prove to be more efficient for the agent and the BG regulation since there are more choices in insulin amounts. Similar to experiment 1, we compare all the DQN algorithms using the same procedure and metrics. We used the same state space as in experiment 1, but the action space now has five actions in it, as described in Section 2.3.1. The only hyperparameter that was changed from experiment 1 was the batch size of 512. The increase in batch size is to compensate for the fact that we could need more data since more actions could complicate the learning process. Table 2 summarizes the results from experiment 2.

Comparable to the results from experiment 1, the DQN algorithm performs similarly to the baseline, with no hypoglycemic events and almost no differences in terms of the TIR (95.23%) and TAR (4.77%). The mean BG per episode (124.46 mg/dL) is also close to the value obtained from baseline (124.00 mg/dL), whereas the standard deviation is slightly higher when using a DQN agent.

The DDQN agent also works similarly to the experiment 1 version, in which actions with high basal rates lead to lower episodic BG levels, μ = 103.29 mg/dL. As a consequence of the lower mean BG per episode, the DDQN extension presents a lower TIR (93.80%) than baseline (95.41%), avoiding hyperglycemic events and scoring the worst TBR (6.20%) in this experiment 2.

For the dueling DQN agent, we obtained the best TIR (97.04%). This agent also decreases TAR (2.96%) compared with baseline (4.59%), reducing the mean BG per episode (113.80 mg/dL) with no hypoglycemic events and slightly higher standard deviation (34.03 mg/dL). Compared to experiment 1, the diversity in the action selection has helped the agent to learn a better usage of the different basal rates included in the action space, proving a successful control of the BG concentrations.

The dueling DDQN method performs worse than the baseline, decreasing the TIR (94.91%) and scoring the worst TAR (5.09%) while still able to avoid hypoglycemia. However, the agent reduces the mean BG per episode (119.61 mg/dL) and obtains the lowest standard deviation (31.60 mg/dL). Compared to experiment 1, the inclusion of more actions in the action space has hindered the learning process and so worsened the performance in terms of the TIR and TAR.

The results in Table 2 reveal that PR DQN presents a worse TIR (93.75%) and TBR (5.60%) than the baseline while reducing the TAR (0.65%). These undesired results are a consequence of the high basal rate actions chosen by the agent, obtaining the closest mean BG per episode (107.04 mg/dL) to the target value. The scenario was the opposite in experiment 1, in which the agent scored the highest TAR and zero TBR.

Noisy DQN presents a lower TIR (94.17%) than the baseline and zero TAR, resulting in a mean BG per episode (109.54 mg/dL) closer to the target value. This agent struggles with controlling the BG levels due to high basal rate choices leading to a high TBR (5.83%), whereas noisy DQN emerged as the best solution for controlling BG in experiment 1. High basal rate actions are a common wrong strategy learned by some of the agents after expanding the action space. With more actions to choose from, the agent might be confused since the added noise in the layers encourages the agent to explore even more than before. This problem might be alleviated by a steeper decay on the ε-greedy action selection. Moreover, sometimes more neurons increase the probability of learning useful information, so a deeper network might help with exploration and exploitation.

Similar to PR DQN and noisy DQN, the strategy learned by categorical DQN is defined by actions with a high basal rate leading to a low mean BG per episode (106.28 mg/dL). These actions result in a very high TBR (6.11%) and zero TAR, with a lower TIR (93.89%) than baseline (95.41%). Compared with experiment 1, the extension of the action space worsens the results for this method, exposing the difficulties of the agents for learning with an expanded action space.

The results from rainbow DQN are even worse than in experiment 1, with the agent giving too high basal rates which lead to very high TBR (6.02%). The TIR (90.56%) is the lowest, whereas the standard deviation (42.50 mg/dL) is the highest among the tested methods, obtaining the worst results from Table 2. In both experiments 1 and 2, rainbow DQN fails to control BG levels as intended.

Experiment 2 has shown that increasing the number of actions does not necessarily improve the performance in the BG control task. The larger the action space, the more difficult the learning process, hindering the decision-making by the agent. DDQN, PR DQN, noisy DQN, categorical DQN, and rainbow DQN agents did not succeed when regulating BG concentrations better than baseline. Only the dueling DQN algorithm improved the results from the baseline, whereas DQN and dueling DDQN obtained very similar results.

**Figure 5:**
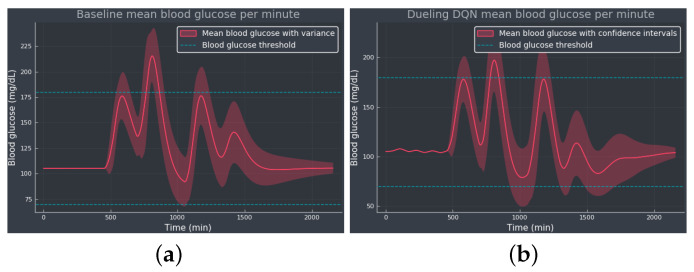
The mean BG per minute with confidence bands (shaded red area), representing the standard deviation, simulated for 100 episodes: (**a**) The baseline using only the optimal basal rate $b^{*}=6.43$ mU/min as the selected action. (**b**) The dueling DQN agent. The blue dotted lines indicate the normoglycemic range fixed at 70–180 mg/dL.

Figure 5 shows the mean BG values obtained from the baseline and dueling DQN agent, considered the most competitive RL approach from the results obtained in Table 2. Comparing both methods, we see how the dueling DQN agent improves the results obtained from the baseline by decreasing the TAR and thus increasing the TIR while avoiding hypoglycemia. Note how the agent regulates the tail of the curve to the optimal BG value, showing that the agent was able to learn the optimal basal rate of our simulated patient. The RL algorithm reduces the mean BG concentrations, working closer to the target value, although slightly increasing the standard deviation.

### 3.3. Experiment 3—Meal Bolus Perturbation

Experiment 3 investigates the ability of a trained agent to deal with skipped meal boluses. The agents are trained following the experimental setup from experiment 1. After training the agents, a similar comparison to that of experiments 1 and 2 was performed, in which the action variance per episode $\sigma_{A}$ was also calculated. The BG data were obtained from 100 simulated episodes, in which meal schedules were generated with a set seed and meal boluses were skipped with a 10% probability. The goal of this experiment is to compare the algorithms’ performances on an unstable meal schedule. Table 3 summarizes the results from experiment 3.

Starting with DQN, the TIR has dropped by roughly 3%, whereas the TAR (8.43%) and the standard deviation of the BG per episode have increased as a consequence of skipping insulin boluses during meals. The variance in the insulin action is very low, almost zero, indicating that the agent chooses the same action most of the time.

The DDQN agent’s performance is not affected by the skipped meal boluses, since the results obtained in experiment 3 are very similar to the results obtained in experiment 1. In this case, the agent presents a moderate variation in the insulin actions, with $\sigma_{A}$ = 4.72 mU/min.

For the dueling DQN, the performance improves in terms of TBR (0.0%) at the cost of decreasing the TIR (92.18%) and increasing the TAR (7.82%). However, the low TBR obtained might be an artifact due to the BG concentrations being higher as a consequence of the skipped meal boluses and thus increasing the TAR.

The dueling DDQN also shows very similar results in both experiments 1 and 3 and was the only algorithm able to reduce the mean BG per episode (123.41 mg/dL) when compared with experiment 1, showing some robustness against skipped meal boluses. This agent also presents the highest insulin action standard deviation per episode (9.06 mU/min), showing a more rapid variation in insulin actions.

For PR DQN, noisy DQN, and categorical DQN, the TIR scores have lowered by approximately 1% and the TAR have slightly increased. Similar to experiment 1, noisy DQN scored the highest TIR (96.20%), with a low $\sigma_{A}$ = 4.50 mU/min suggesting that less insulin action variation might be beneficial for the controlling process.

Rainbow DQN seems to be unaffected by the meal disturbances at first glance. With the lowest TBR score, rainbow DQN nearly obtained the same results as experiment 1.

## 4. Discussion

In this work, the current state-of-the-art DQN algorithms have been tested and evaluated for the task of controlling the BG levels in a simulated T1D patient. These algorithms were compared to a baseline, where only the optimal basal rate was given to the patient. Concretely, three experiments were conducted. Goals with TIR vary from person to person and may depend on the type of medication they use, type of diabetes, diet, health, age, and risk of hypoglycemia [52]. Generally, any patient suffering from diabetes should spend as much TIR as possible, which is the main goal of our first experiment. The results from this experiment show the potential of DQN algorithms to successfully regulate BG levels in T1D, with dueling DDQN performing better than the standard treatment and the noisy DQN agent achieving the highest TIR.

Experiment 2 evaluates how a larger action space affects the performance of the agents. Our experiments show that the DQN algorithms perform better with an action space with three actions, rather than an action space with five actions. The extended action space is hindering the training process of the agents, resulting in undesirable high insulin action tendencies. In this setting, DQN and dueling DDQN performed close to the baseline, whereas dueling DQN was the only algorithm able to outperform it. Compared to the smaller action space, the experiments showed more promising results, with DQN and categorical DQN performing similarly to the baseline and dueling DDQN and noisy DQN outperforming it.

A final experiment was conducted, skipping meal boluses at random for an already trained agent. The goal of this test is to gain a deeper understanding of which algorithm performs best when the meal schedule is more unstable. When skipping meal boluses, we found that the overall TIR was slightly lowered whereas the TAR was slightly higher. The TBR was virtually unchanged, suggesting that this experiment led to more algorithms failing during the BG control task and so neither adapting nor generalizing to the lack of meal boluses. Only the dueling DDQN agent was able to obtain a similar TIR while reducing mean BG per episode during experiment 3, showing some robustness against skipped meal boluses.

Given the current experimental setup, further experiments need to be carried out to fully validate DQN as a realistic algorithm for the AP, since the extent of these experiments is not sufficient to claim that the DQN algorithms are beneficial in a complete and general sense.

## 5. Conclusions and Future Work

In this work, we have shown that some state-of-the-art DQN algorithms outperform standard base bolus treatment in our experiments. The most competitive results were obtained by noisy DQN for experiments 1 and 3 and by dueling DQN for experiment 2. These DQN agents were able to cope with both carbohydrate counting errors and to a certain degree skipped boluses. Therefore, we consider this work a strong proof of concept for the use of DQN algorithms in the AP framework. However, most of the algorithms did not perform better than the baseline when controlling BG levels in experiment 2, indicating that there is room for improvement in both the algorithm’s implementation and the environment setup.

Experimenting with different types of NN architectures might help to alleviate the training problems associated with the larger action space, leading to better learning for future work. One step further in this research direction would be to test policy gradient algorithms, allowing the use of a continuous action space instead of a discrete one. Moreover, it would also be worth it to test different state spaces; for example, use the last 24 h of BG and insulin data.

Due to the fact that T1D is a well-studied disease and multiple treatment strategies already exist, there is a lot of domain knowledge that gets lost in our experiments. An obvious research direction is including domain knowledge into the RL framework for T1D; for example, through the reward function.

## Figures and Tables

**Table 1:** Experiment 1—TIR, TAR, and TBR of the mean BG per minute of 100 episodes for the different DQN extensions. μ is the mean BG per episode and σ is the standard deviation of the BG per episode. Results better than baseline are written in blue text, with the best results highlighted in **blue bold text**. Results worse than baseline are written in red text. Note that in the TBR column, there are multiples of the same result, hence they are not highlighted.

Algorithm	TIR (%)	TAR (%)	TBR (%)	μ (mg/dL)	σ (mg/dL)
Baseline	95.41	4.59	0.0	124.00	33.84
DQN	95.05	4.95	0.0	125.04	35.21
DDQN	92.82	1.90	5.28	**111.67**	33.31
Dueling DQN	93.33	0.45	6.25	124.32	32.24
Dueling DDQN	96.71	3.29	0.0	126.92	32.32
PR DQN	94.35	5.65	0.0	122.63	33.26
Noisy DQN	**97.04**	2.96	0.0	116.10	**31.74**
Categorical DQN	95.23	4.77	0.0	125.25	34.68
Rainbow DQN	94.35	**0.0**	5.65	100.66	32.20

**Table 2:** Experiment 2—TIR, TAR, and TBR of the mean BG per minute of 100 episodes for the different DQN extensions using the expanded action space. μ is the mean BG per episode and σ is the standard deviation of the BG per episode. Results better than baseline are written in blue text, with the best results highlighted in **blue bold text**. Results worse than baseline are written in red text. Note that in the TBR column, there are multiples of the same result, hence they are not highlighted.

Algorithm	TIR (%)	TAR (%)	TBR (%)	μ (mg/dL)	σ (mg/dL)
Baseline	95.41	4.59	0.0	124.00	33.84
DQN	95.23	4.77	0.0	124.46	34.88
DDQN	93.80	0.0	6.20	103.29	31.65
Dueling DQN	**97.04**	2.96	0.0	113.80	34.03
Dueling DDQN	94.91	5.09	0.0	119.61	**31.60**
PR DQN	93.75	0.65	5.60	**107.04**	33.70
Noisy DQN	94.17	0.0	5.83	109.54	32.98
Categorical DQN	93.89	0.0	6.11	106.28	32.28
Rainbow DQN	90.56	3.43	6.02	118.35	42.50

**Table 3:** Experiment 3—TIR, TAR, and TBR of the mean BG per minute of 100 episodes for the different DQN extensions including skipped meal boluses. μ is the mean BG per episode, σ is the standard deviation of the BG per episode, and $\sigma_{A}$ is the insulin action standard deviation per episode. Results better than baseline are written in blue text, with the best results highlighted in **blue bold text**. Results worse than baseline are written in red text. Note that in the TBR column, there are multiples of the same result, hence they are not highlighted.

Algorithm	TIR	TAR	TBR	μ (mg/dL)	σ (mg/dL)	$\sigma_{A}$ (mU/min)
Baseline	94.49%	5.51%	0.0%	125.95	36.47	0.0
DQN	91.57%	8.43%	0.0%	129.54	39.01	$8.88\times 10^{-16}$
DDQN	93.15%	2.45%	4.40%	**113.33**	35.59	4.72
Dueling DQN	92.18%	7.82%	0.0%	126.11	35.65	5.85
Dueling DDQN	96.16%	3.84%	0.0%	123.41	34.43	9.06
PR DQN	93.61%	6.39%	0.0%	123.50	34.41	6.92
Noisy DQN	**96.20%**	3.8%	0.0%	117.73	34.97	4.50
Categorical DQN	94.49%	5.51%	0.0%	125.95	36.47	8.88
Rainbow DQN	94.21%	**0.0%**	5.79%	101.41	**33.57**	7.91

## Data Availability

The data presented in this study are available in the article.
